# Quantifying homicide trends in Australia: a methodological caution

**DOI:** 10.5249/jivr.v4i1.176

**Published:** 2012-01

**Authors:** Samara McPhedran, Jeanine Baker

**Affiliations:** ^*a*^International Coalition for Women in Shooting and Hunting (WiSH), New South Wales, Australia

Reducing the incidence of assault-related deaths remains an ongoing interest for injury prevention practitioners. In Australia, and elsewhere, an area of particular attention has been quantifying the incidence of firearm homicide (and tracking potential method substitution) following legislative change. In Australia, the majority of epidemiological studies in this field rely on Australian Bureau of Statistics (ABS) data. However, it has recently been noted by the Australian Institute of Criminology (AIC) that the ABS have been ‘undercounting’ firearm homicides since the year 2004. Specifically “…coding procedures used since 2004 (related to an increase in the number of open coroners cases) have resulted in an under-counting of firearm deaths due to assault (i.e. firearm homicide)”.^1^ABS data do not take into account coroners cases that were ‘open’ at the time of annual ABS data collation.

The ABS under-counting issue has previously been flagged in relation to suicide.^2-4^Recent cross-checks of ABS suicide data against other sources (for example, the National Coronial Information System)^2,3^suggest that the actual number of suicides from the early 2000s onwards may be in the order of between 200 and 400 deaths per year higher than ABS data indicate. It has been suggested that at least part of the apparent decline in suicide rates since the mid 1990s may in fact be due to under-counting, rather than representing a ‘real’ decline.^2^

Discrepancies between actual and recorded numbers of deaths have clear implications for the development of robust, evidence-based injury prevention policy. It is concerning, therefore, that the under-counting issue for suicides also appears to apply to homicide data. This implies that the annual number of firearm and non-firearm homicides may be higher than thought. Given the relatively low number of homicides each year in Australia, a small number of ‘uncounted’ deaths could have substantial impacts on observed homicide rates. This in turn could impact on research findings and the evidence base used to inform policy development. While under-counting is unlikely to seriously affect studies of firearm and non-firearm homicide that use pre-2005 data,^5^it has potential to significantly alter studies that include data from 2004 onwards. It is recommended that the under-counting issue be investigated as a matter of priority, with ABS data revised as appropriate. In the interim, we suggest extreme caution when examining data on deaths due to assault from 2004 onwards.
